# Forehead Ultrasound Anatomy: The Current Debate and a Way to Consensus

**DOI:** 10.1093/asj/sjae186

**Published:** 2024-08-27

**Authors:** Stella Desyatnikova, Rosa Sigrist, Ximena Wortsman

*It is a myth that the success of science in our time is mainly due to the huge amounts of money that have been spent on big machines. What really makes science grow is new ideas, including false ideas.*

—Karl Popper

Several recent papers in *Aesthetic Surgery Journal* have explored forehead ultrasound anatomy, reflecting growing interest in ultrasound as a tool for research and clinical practice.^1-4^ However, there is controversy surrounding the identification of individual anatomic layers by ultrasound. What are the reasons behind this debate, and how can we move towards a consensus?

Facial ultrasound is an emerging field, bridging dermatologic ultrasound and aesthetic medicine. Due to its operator-dependent nature, accurate image interpretation requires proper scanning techniques and a deep understanding of both fields. Forehead ultrasound typically shows a consistent sequence of hypoechoic (dark) and hyperechoic (white) layers (Figures 1, 2). The apparent simplicity of these images is misleading and has led to errors and inconsistencies in the literature. Inconsistency begets further confusion.

**Figure 1.**
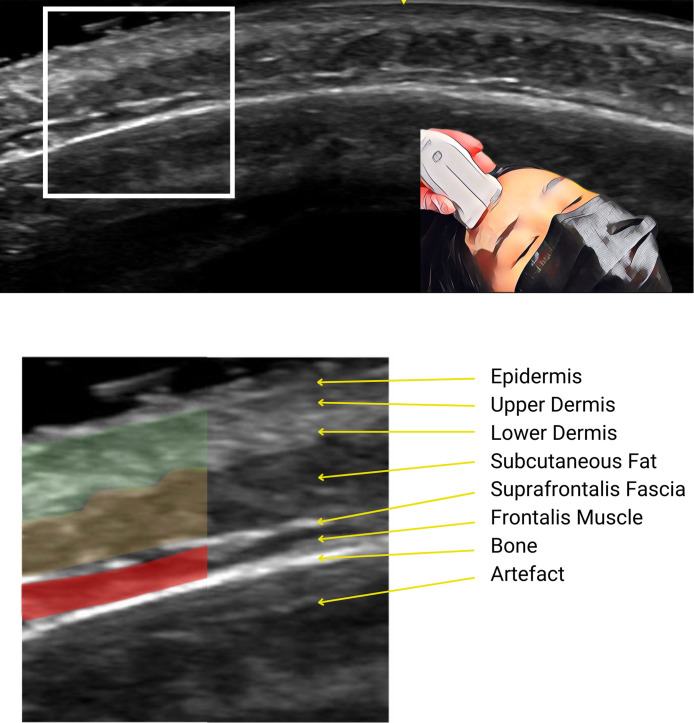
Forehead ultrasound anatomy, of a 24-year-old female, performed with the probe positioned as shown in the inset. Tissue layers are identified in the lower image, with each color representing a distinct tissue layer (green, upper and lower dermis; orange, subcutaneous fat; red, frontalis muscle).

**Figure 2.**
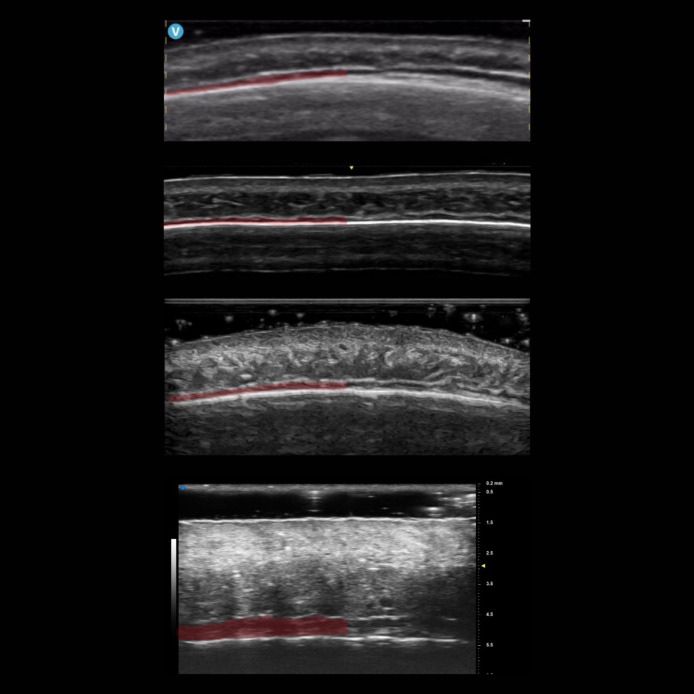
Forehead ultrasound images taken with different devices. Top to bottom: portable linear 12 MHz probe, linear 4-20 MHz probe, linear 6-24 MHz probe, and ultrahigh-frequency 70 MHz probe. The frontalis muscle is marked in red.

Previous papers, including recent *Aesthetic Surgery Journal* articles,^1-4^ have identified the same layers differently. One of the main disagreements is whether the thick hypoechoic layer in the middle represents frontalis muscle or subcutaneous fat. To address this controversy, it helps to review established concepts.

## ULTRASOUND CONCEPTS

1. The appearance of tissues on ultrasound is consistent.
2. Subcutaneous fat is hypoechoic, with linear hyperechoic septae. Muscle is hypoechoic as well. Bone and fascial layers are hyperechoic.^5-7^
3. The dermis is a hyperechoic layer that frequently appears as 2 bands: the hypoechoic upper (subepidermal low-echogenicity band) and the hyperechoic lower. The subepidermal low echogenicity band develops over time and becomes more prominent with photoaging changes (elastosis).^5-7^ It should not be confused with subcutaneous fat.
4. Ultrasound images display the accurate position of structures, allowing for the measurement of depth and thickness of objects. However, ultrasound images may be affected by artifacts.
5. The layers appear similar, albeit with small variations, on different devices (Figure 2).

## ANATOMIC CONCEPTS

1. Forehead layers include the epidermis, dermis, subcutaneous fat, frontalis muscle surrounded by fascia, loose connective tissue with fat, and periosteum.
2. The epidermis, dermis, and subcutaneous fat are continuous across the face, including the forehead, temples, and cheeks.
3. The frontalis muscle extends laterally to the temporal crest and may end slightly lateral or medial to it. It does not continue into the temple or into the cheek area. A midline attenuation of the frontalis muscle in the upper forehead is present occasionally and can be appreciated on ultrasound examination.
4. In surgery, anatomic dissections, and histologic images,^4^ we usually see a thick middle layer of subcutaneous fat, and a thin deep layer of the frontalis muscle.
5. Thin loose connective tissue layer gets thicker in the inferolateral portion and is known here as retro-orbicularis oculi fat.

**Figure 3.**
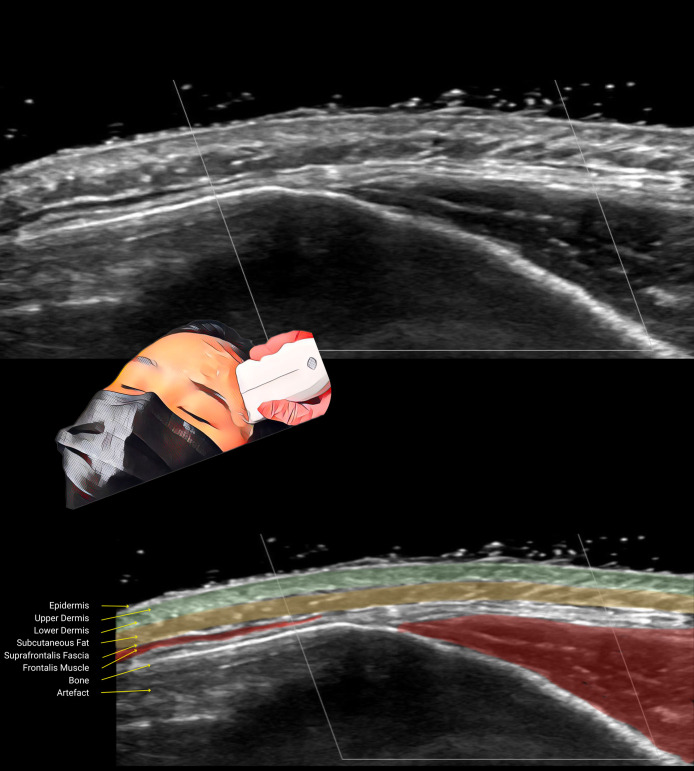
Imaging in the diagonal plane across the temporal crest, of 24-year-old female, performed with the probe positioned as shown in the inset. Note the hypoechoic frontalis muscle ending at the level of temporal crest while the dermis and subcutaneous fat layers continue into the temple and beyond. The frontalis and temporalis muscles are marked in red, the subcutaneous fat layer is marked in yellow, and the dermis (upper and lower) is marked in light green.

**Figure 4.**
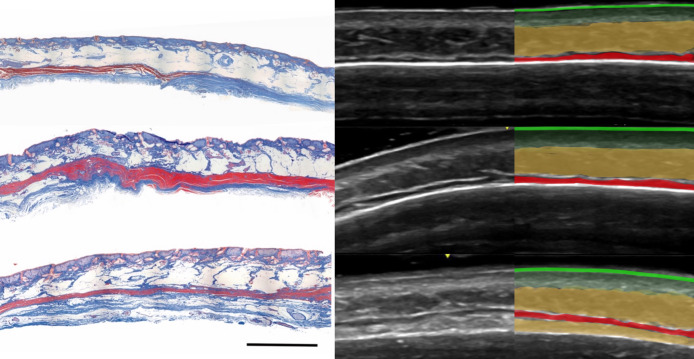
Forehead histology and corresponding ultrasound images, in the axial plane, taken at similar levels—upper, middle, and lower forehead. The frontalis muscle layer is red on histology, hypoechoic (dark) on ultrasound, and marked in red on the color diagram. On the ultrasound image, the subcutaneous fat layer is marked in yellow, the dermis (upper and lower) is marked in light green, and the epidermis is marked as a bright green line. In the lower forehead, deep fat (retro-orbicularis oculi fat) is marked in yellow. Note the similar frontalis muscle position on histology and ultrasound. In the lower part of the forehead, the muscle is more superficial due to a layer of retro-orbicularis oculi fat. The histologic image is reproduced with permission from Angrigiani et al.^4^

With this information in mind, we can review the images again, including panoramic images across the forehead and temple (Figure 3; Video), and definitively identify the layers as follows:

1. A thin hyperechoic line, about 0.1 mm thick, representing the epidermis.
2. Hypoechoic and hyperechoic layers with a combined thickness of about 1.0 mm, continuous into the temples (Figure 3; Video), consistent with the definition and thickness of the dermis.
3. A thick hypoechoic layer below the dermis, continuous into the temples and cheeks (Figure 3; Video). The thickness of this layer, its location just below the dermis, and its extension beyond the forehead, confirm it as subcutaneous fat and not frontalis muscle.
4. A thin hyperechoic suprafrontalis fascia.
5. A hypoechoic layer, 0.5 to 1.0 mm thick (Figures 1-4; Video), located deep above the bone superiorly and more superficially in the inferolateral forehead. Ultrasound images show this layer ending above the temporal crest, while other layers continue into the temple. This, combined with echogenicity, thickness, and position above the bone, confirms this layer as the frontalis muscle, and corresponds to histologic images.^4^
6. A loose connective tissue layer seen in the inferolateral forehead, as dense retro-orbicularis oculi fat with increased echogenicity (Figures 3, 4; Video).
7. A hyperechoic periosteum and frontal bone, with an acoustic shadow and artifacts of mirror-imaging and reverberation below.

We hope this description aids in achieving consensus concerning interpretation of forehead ultrasound. Ultrasound imaging provides the advantage of real-time visualization of anatomy, but incorrect interpretation can result in adverse clinical outcomes, such as poor neuromodulator results and vascular adverse events.

Further research and rigorous scientific discourse are essential to ensure the safety and efficacy of aesthetic procedures.
